# Supplementary material for: MetaRibo-Seq measures translation in microbiomes
Source: Nat Commun. 2020 Jun 29;11:3268. doi: 10.1038/s41467-020-17081-z (PMC7324362; doi:10.1038/s41467-020-17081-z)
Supplement: Supplementary file 10 — Supplementary Data 7 [file 41467_2020_17081_MOESM10_ESM.zip › File2/Confidence_High_Taxonomy/282094_out.krona.html]

Javascript must be enabled to view this page.

members
magnitude
magnitudeUnassigned
count
unassigned
taxon
rank

282094\_out

3

3
superkingdom
2

phylum
3
1224

class
3
28216

80840
order
3

family
3
119060

1822464
3
genus

species
3

SRS023914\_contig\_number\_29260SRS098571\_contig\_number\_10483SRS1041145\_contig\_number\_30856
2211211
